# Automated Detection of Myocardial Infarction and Heart Conduction Disorders Based on Feature Selection and a Deep Learning Model

**DOI:** 10.3390/s22176503

**Published:** 2022-08-29

**Authors:** Mohamed Hammad, Samia Allaoua Chelloug, Reem Alkanhel, Allam Jaya Prakash, Ammar Muthanna, Ibrahim A. Elgendy, Paweł Pławiak

**Affiliations:** 1 Department of Information Technology, Faculty of Computers and Information, Menoufia University, Shibin El Kom 32511, Egypt or; 2 Department of Information Technology, College of Computer and Information Sciences, Princess Nourah bint Abdulrahman University, P.O. Box 84428, Riyadh 11671, Saudi Arabia; 3 Department of Electronics and Communication, National Institute of Technology Rourkela, Rourkela 769008, India; 4 Department of Applied Probability and Informatics, Peoples’ Friendship University of Russia (RUDN University), 117198 Moscow, Russia; 5 Department of Computer Science, Faculty of Computers and Information, Menoufia University, Shibin El Kom 32511, Egypt; 6 Department of Computer Science, Faculty of Computer Science and Telecommunications, Cracow University of Technology, Warszawska 24, 31-155 Krakow, Poland; 7 Institute of Theoretical and Applied Informatics, Polish Academy of Sciences, Baltycka 5, 44-100 Gliwice, Poland

**Keywords:** CNN, conduction disorders, deep learning, feature selection, myocardial infarction, SVM

## Abstract

An electrocardiogram (ECG) is an essential piece of medical equipment that helps diagnose various heart-related conditions in patients. An automated diagnostic tool is required to detect significant episodes in long-term ECG records. It is a very challenging task for cardiologists to analyze long-term ECG records in a short time. Therefore, a computer-based diagnosis tool is required to identify crucial episodes. Myocardial infarction (MI) and conduction disorders (CDs), sometimes known as heart blocks, are medical diseases that occur when a coronary artery becomes fully or suddenly stopped or when blood flow in these arteries slows dramatically. As a result, several researchers have utilized deep learning methods for MI and CD detection. However, there are one or more of the following challenges when using deep learning algorithms: (i) struggles with real-life data, (ii) the time after the training phase also requires high processing power, (iii) they are very computationally expensive, requiring large amounts of memory and computational resources, and it is not easy to transfer them to other problems, (iv) they are hard to describe and are not completely understood (black box), and (v) most of the literature is based on the MIT-BIH or PTB databases, which do not cover most of the crucial arrhythmias. This paper proposes a new deep learning approach based on machine learning for detecting MI and CDs using large PTB-XL ECG data. First, all challenging issues of these heart signals have been considered, as the signal data are from different datasets and the data are filtered. After that, the MI and CD signals are fed to the deep learning model to extract the deep features. In addition, a new custom activation function is proposed, which has fast convergence to the regular activation functions. Later, these features are fed to an external classifier, such as a support vector machine (SVM), for detection. The efficiency of the proposed method is demonstrated by the experimental findings, which show that it improves satisfactorily with an overall accuracy of 99.20% when using a CNN for extracting the features with an SVM classifier.

## 1. Introduction

A heart attack (the scientific name of which is myocardial infarction (MI)) is a condition in which there is a blockage or narrowing in one or more of the coronary arteries that supply the heart muscle. The main cause of this disease is atherosclerosis [1]. The hardening process begins at a relatively young age and develops gradually over the years. This is a complex process involving various blood cells, cholesterols, proteins, and hormones, which together cause the formation of a hardening plaque in the walls of blood vessels. This plaque develops from a thin layer to become a mass of tissue that grows into the artery lumen and impedes blood flow through it. The causes of MI and risk factors are as follows [2]:

- Coronary artery disease.
- Hypoxia.
- Taking drugs or certain toxic chemicals, such as cocaine.

The hazard of this type of health problem is that it typically strikes the patient unexpectedly, necessitating immediate action to limit the crisis for fear of death or severe cardiac damage. Therefore, early diagnosis of an MI can be critical for treatment [3]. An electrocardiogram (ECG) is an examination that enables the progression of an electrical wave that regulates the work of the heart muscle. In a typical pacemaker, this electrical wave goes through the atria and forces them to contract, assisting blood flow from the atria to the ventricles. The electrical signal then travels to the heart chambers via unique conducting fibers, causing them to contract, resulting in blood flow from the left ventricle to the bodily tissues via the aorta and blood flow from the right ventricle to the lungs. The existence of abnormalities in the creation and conduct of the electrical wave, which may be produced by disturbances in the heart conduction system, can be detected via an ECG examination [4]. Moreover, changes in the ECG may be an indicator of the presence of MI, whether they are recent or old. In short, the ECG processing methodology can help detect the most common heart diseases, such as arrhythmias, coronary heart disease, and heart attacks. However, manual evaluation of ECG signals is time consuming and tedious. Therefore, accurate detection of MI in the medical field is very critical for timely diagnosis by doctors and clinicians. As a result, researchers are trying to develop an accurate methodology for the automatic detection of MI.

### Related Work and Motivation

As mentioned before, the previous methods are classified into two categories, namely, machine learning and deep learning approaches. The different machine learning techniques available in the literature are the support vector machine (SVM) and K-nearest neighbor (KNN) [5], Fourier decomposition method (FDM) with SVM [6], etc. However, convolutional neural networks (CNNs), recurrent neural network (RNNs) [7], residual networks [8], and capsule networks [9] are also utilized to detect different types of beats.

However, the authors have only included approaches based on deep learning that are relevant to the scope of the work presented. Furthermore, several recent review studies in this field showed that almost all recent works in this field have focused on deep learning [8,10,11,12,13].

Smigiel et al. [14] developed three deep learning methods to automatically classify primary ECG signals. The first method was based on CNN, the second method was based on SincNet, and, finally, the last method was based on CNN with entropy-based features. They worked on five superclasses from the PTB-XL dataset and obtained the best overall accuracy of 76.50% using a the CNN with entropy. In other studies, Smigiel et al. [8] also automatically classified the ECG signals based on deep learning techniques and R-peak detection. They worked on five superclasses from the same database (PTB-XL) and obtained the best overall accuracy of 76.20%. Pałczynski et al. [11] determined the applicability of using Few-Shot Learning (FSL) for the classification of ECG signals. They extracted the QRS complex from the ECG signals and used a deep CNN for classification. They worked on the PTB-XL database on the five superclasses and obtained the best overall accuracy of 79%. Prabhakararao and Dandapat [12] designed a method for multi-class arrhythmia classification based on a CNN ensemble. They used data augmentation techniques and preprocessing to reduce the computational burden and for baseline artifact removal. They evaluated their method on the 12-lead of the PTB-XL database on the five superclasses and obtained an overall accuracy of 85.65%. Zhang et al. [13] presented a multi-lead fusion method for multi-class arrhythmia classification. They worked on the five superclasses from the PTB-LX database and obtained an overall accuracy of 93.10%. All of these previous methods [8,11,12,13,14] obtained low accuracy when using the five superclasses for classification. The proposed method achieved the highest accuracy compared with these methods on the five superclasses. Table 1 shows a comparison of related works based on different criteria.

There are various artificial intelligence (AI) approaches that are employed for the detection of MI based on the analysis of ECG signals [5,6,19]. These approaches are categorized into two common approaches, namely, machine learning [7,20,21] and deep learning approaches [17,18,22]. Deep learning methods are thought to be more reliable than typical machine learning methods, especially when dealing with large amounts of data. Furthermore, the multi-layer structure of deep learning techniques provides tools for effective feature interpretation and pattern recognition, which are critical when classifying large unstructured datasets. Despite their superior features, typical deep learning networks are widely acknowledged to have several drawbacks, which include the following:

- Misclassification in some high-inter-class-imbalance cases.
- Increasing the data over-fitting as a result of depleting the datasets, which reduces detection accuracy and, particularly, sensitivity.
- Obtaining low accuracy when implementing these methods in real-time applications.
- Requiring the detection of the QRS complex.
- Using complex signal processing methods and inefficient MI detection methods.

Consequently, this work is aimed toward overcoming the above drawbacks via a novel technique based on deep learning approaches for MI detection. Recently, deep learning approaches have shown success in many applications, such as pattern recognition applications [23,24], internet of things (IoT) applications, medical applications [25], etc.

In this paper, we first filter the ECG signals to eliminate the noise. Then, we propose a deep learning model based on a convolutional neural network (CNN) in order to extract the deep features from the input signal. After that, we optimize and select the features from the convolutional layers. Finally, the selected features are fed to an external classifier, such as an SVM, for MI detection. According to the results of the analysis and the investigation into the PTB-XL database [26], the proposed method outperforms recent deep learning methods. This paper has the following main contributions:

- The deep features are extracted from the fine-tuned CNN architecture, which eliminates the requirement of handcrafted feature extraction techniques. The whole end-to-end system can also be called explainable artificial intelligence (XAI).
- A new customized activation function is designed and the convergence of the function is tested through experimentation. The proposed activation function is fast compared to the regular activation functions, such as the sigmoid function.
- The network is validated on a large publicly available dataset, i.e., PTB-XL, which allows the detection of crucial cardiac disorders, such as MI, CD, HYP, and STTC. This is the major contribution of the work, as more than 90% of the literature is based only on MIT-BIH and PTB.
- The proposed algorithm is able to achieve good results in classification tasks, which is an important component of the development of automated computer-aided systems for the detection of MI and CDs. The proposed method allows the user to understand the cause of the decision of the machine learning by analyzing feature maps extracted by the deep learning algorithm.
- The proposed end-to-end ECG beat classification system enables human users to understand and effectively manage emerging decisions in intensive care units (ICUs).

## 2. Methodology and Dataset

The detailed methodology and the dataset (PTB-XL) used to evaluate the performance of the proposed network architecture are described in this section. The dataset includes a significant portion of healthy records, as well as a wide range of diagnostic classes. Beyond the demonstration of the exceptional performance of closed-source algorithms on customized datasets with restricted access, there are at least two significant barriers preventing further advancement in this area: (1) the non-availability of large publicly accessible datasets for proper training; (2) the absence of clear evaluation methods for these algorithms. We hope to address both problems and close this research gap by combining machine and deep learning techniques. PTB-XL is a large dataset with an outstanding variety, and it is distinguished by its signal quality. Its rich coverage of pathologies, many different co-occurring diseases, and a large number of healthy control samples is rarely found in clinical datasets. Therefore, PTB-XL is a great choice for training and testing algorithms in the real world, where machine or deep learning algorithms have to work reliably no matter the recording conditions or the data quality. A detailed description of the beats in the database is given in Section 2.1.

### 2.1. ECG Dataset Description

In this study, we employed the PTB-XL electrocardiography dataset [26], which is an updated version of the PTB database and the most common dataset used for MI detection in the literature [13,15,16,17,18,22]. This dataset consists of 21,837 records with a length of 10 s collected from 18,885 mixed individuals (men and women). For each individual, there are several ECG records (ranging from one to five records). The records in this dataset include 12 conventional leads with reference electrodes on the right arm. The signals have a 16-bit resolution and are digitized at 500 samples per second. The dataset is labeled with two classes, namely, the superclass and subclass. A detailed description of the superclass and subclass is given in [26]. In this paper, we worked on the superclass, which is classified into the normal class (NORM) and 4 diagnostic classes, namely, MI, conduction disturbance (CD), hypertrophy (HYP), and ST/T change (STTC). In PTBXL data, ECG signals are not annotated beat by beat; experts annotate the whole 10-second-duration signal with multiple annotations. When one signal contains multiple labels, it is very difficult to classify it. Therefore, we chose single-labeled data only. Table 2 shows the number of beats in the original dataset with and without overlapping. The detailed splitting of the data for the experimentation is shown in Table 3.

### 2.2. MI Methodology

**Figure 1:**
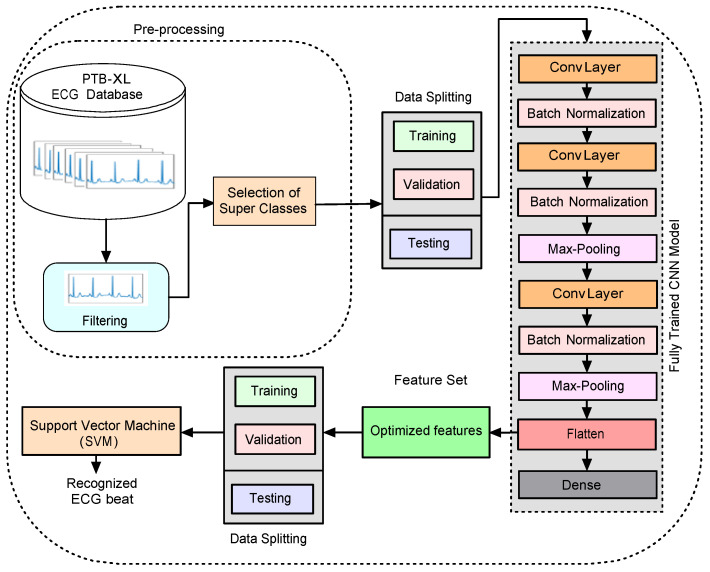
General diagram of the proposed method.

The general block diagram of the proposed method for the detection of MI and CDs is shown in Figure 1. As shown in the figure, we find that the proposed system comprises the following stages: a signal preparation and pre-processing stage, a deep feature extraction stage, a feature selection stage, and, finally, the detection stage. The detailed description of these stages is as follows.

#### 2.2.1. ECG Signal Pre-Processing

First, the input ECG signals were down-sampled to 100 Hz, and a 10-s ECG signal was present in each row of a particular class. ECG signals were then caught and recorded by skin electrodes, making them susceptible to contamination from numerous sorts of artifacts or noise. Therefore, the down-sampled ECG signals were filtered using a band-pass filter of 0.1–100 Hz to eliminate the noise. Then, we selected the ECG signals from the superclasses and split the data into training, validation, and testing sets using a 10-fold cross-validation technique [27].

**Figure 2:**
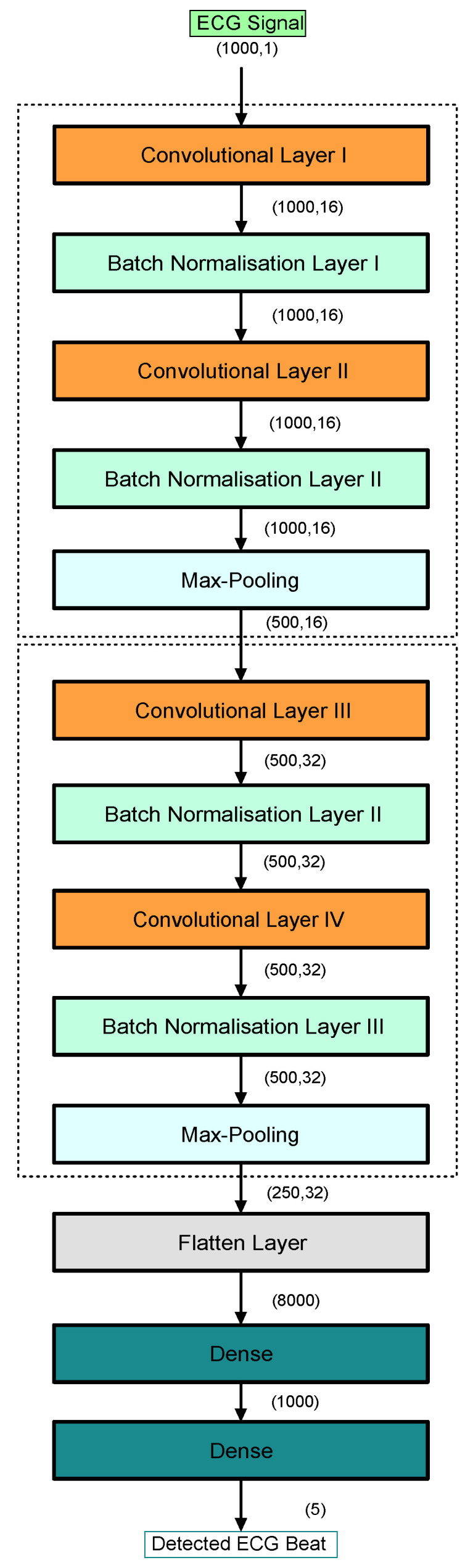
The architecture of the deep learning model.

Figure 2 shows the network architecture of the proposed CNN model. As shown in the figure, the proposed model consists of 14 layers, which are categorized as 4 Conv_layers, 2 max-pooling layers, 4 batch normalization layers, a flatten layer, and 2 dense layers. The model summary (output shape and parameter numbers) is shown in Figure 2. The max-pooling layer is important for suppressing the convolved feature’s dimensions. This layer removes all noisy activations while also performing de-noising and dimension reduction. The dense layer is frequently used to obtain nonlinear combinations of high-level properties from the output of the convolutional layer.

The proposed CNN model was used to extract the deep features of the input ECG signals. In particular, we selected the output of one layer for the feature extraction. Here, we selected the output of a dense layer as the feature for the proposed method with a dimension of 1 × 1000. We selected this layer because it provided a one-dimensional feature set (F1 to F1000 for each ECG signal row). In addition, we obtained the highest accuracy compared with the other layers when using the output of this layer as a feature extractor. Moreover, as the ECG signals had a duration of 10 s, they had many beats in them, which required more depth in the network in order to extract complex features. Finally, we fed the selected features to the SVM classifier for classification.

The activation function used in this model was customized. We employed a new activation function such that the convergence was fast compared to a regular activation function, such as a sigmoid. The equation of the proposed activation function is as follows:

(1) $$y=f\left(x\right)=\frac{1}{1+10^{-x}}$$

**Figure 3:**
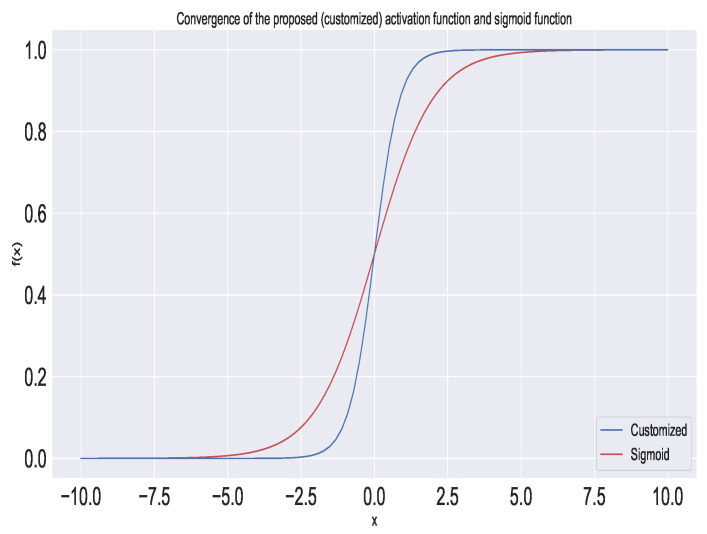
Convergence of the proposed (customized) activation function and the sigmoid function.

The authors compared the proposed activation function with the sigmoid activation function, as shown in Figure 3. In the figure, we can see that the proposed activation function was faster in convergence than the sigmoid function. The major advantages of the proposed activation function are as follows:

- The test loss is small compared to those of conventional activation functions.
- There was faster initial convergence, which decreased the training time of the network.
- There was greater convergence stability over a larger range of learning rates.
- Deep neural networks can be trained much faster than before. High learning rates regularize the network, requiring a reduction of all other forms of regularization to maintain a balance between under-fitting and over-fitting.

The performance of the proposed network with various activation functions is depicted in Table 4.

#### 2.2.2. SVM for MI Detection

Classifiers are used to divide a batch of data into classes. This is a method of mapping input data into a certain class by using an algorithm. In this study, the selected features from the proposed deep CNN model were classified using an SVM. An SVM is a classification technique that can be used to solve problems such as data classification and regression. An SVM uses a hyperplane for two separate classes when deciding on the best separating hyperplane for linearly classifying data [28]. The hyperplane’s expression is given as follows:

(2) $$f\left(x\right)=w^{T}x+b$$

where *x* is the input, $w^{T}$ is the *W* transpose, *w* is a vector normal to the hyperplane, and *b* is an offset. In problems where more than two classes are present for classification (as in this study), a multi-class SVM classifier is used. For multi-class classification using an SVM, several methods are used, which are:

- The one-vs.-one (OVO) approach.
- The one-vs.-all (OVA) approach.

One vs. rest is a heuristic technique for applying binary classification algorithms for multi-class classification. One vs. rest is also known as one vs. all or OVA. The multi-class dataset is divided into various binary classification issues. The most accurate model is used to make predictions after each binary classification problem has been trained with a binary classifier. With this method, each model must forecast a probability of class membership or a score that resembles probability. The class index with the highest score, which is known as the argmax, is then used to predict a class. In this paper, the OVA classification approach is used to classify the ECG signals into NORM and 4 diagnostic classes, namely, MI, CD, HYP, and STTC. The hyper-parameter settings used to implement the SVM in this study are shown in Table 5.

## 3. Computational Results and Discussion

A Python environment was used to implement the experiments in this paper, which made use of the keras, tensorflow, matplotlib, and pandas libraries. The desktop workstation used in this study had the following specifications: The RTX 2060 graphics card had six gigabytes of GDDR6 memory and 32 GB of RAM. We performed pre-processing of the input ECG signals. After that, the pre-processed signals were fed to the proposed deep CNN for extraction of the deep features. Finally, the classification task was performed using an SVM. We applied a 10-fold cross-validation approach in order to evaluate the proposed method. The PTB-XL dataset [26] was employed; this is the expanded version of the PTB dataset [29], which is the most widely used MI signal database in the field of ECG classification research. The average classification time when running the proposed method was 0.2553 s.

### 3.1. Performance Metrics for Evaluating the Proposed Method

The effectiveness of the proposed method was evaluated using four statistical parameters, i.e., precision (*Pre*), recall (*Rec*), accuracy (*Acc*), and the comprehensive *F*-score metric. Rec is also called sensitivity (*Sen*). A detailed explanation of these metrics is available in [12]. The following formulae were used to calculate the performance parameters of the system:

(3) $$Precision\left(Pre\right)=\frac{Sum\;of\;all\;True\;Positives}{Sum\;of\;allTrue\;Positives+Sum\;of\;all\;False\;Positives}=\frac{TP}{TP+FP}$$

(4) $$Recall\left(Rec\right)=\frac{Sum\;of\;all\;True\;Positives}{Sum\;of\;allTrue\;Positives+Sum\;of\;all\;False\;Negatives}=\frac{TP}{TP+FN}$$

(5) $$OverallAccuracy\left(OAcc\right)=\frac{No\;of\;correctly\;detectedinstances}{Total\;number\;of\;instances}=\frac{TP+FN}{TP+TN+FP+FN}$$

(6) $$F1-score\left(F\right)=\frac{2*Precision*Recall}{Precision+Recall}=\frac{TP}{TP+\frac{1}{2}\left(FP+FN\right)}$$

where the true positive (*TP*) rate is concerned, this refers to the number of relevant cases that the model properly identified as relevant. The true negative (*TN*) rate is the proportion of outcomes in which the model predicted the negative class accurately. The false positive (*FP*) rate is the number of irrelevant instances that were wrongly recognized as relevant by the model. The false negative (*FN*) rate refers to the number of relevant events that the model wrongly labeled as irrelevant.

We usually have two classes in binary classification, which are often referred to as “positive” and “negative”, and we tried to predict the class for each sample. In a typical multi-class classification problem, we must classify each sample into one of N different classes. Precision is calculated in an imbalanced classification problem with more than two classes as the sum of true positives across all classes divided by the sum of true positives and false positives across all classes. In imbalanced classification problem with more than two classes, recall is calculated as the sum of true positives across all classes divided by the sum of true positives and false negatives across all classes. Maximizing precision will reduce the number of false positives, while maximizing recall will reduce the number of false negatives.

### 3.2. Comparative Analysis

Two scenarios were employed to evaluate the proposed model. Initially, we trained the proposed deep learning architecture as an end-to-end model with the PTBXL data and then performed the classification. Second, we extracted and selected the features from the flatten layer and fed these features to the SVM to train and test the dataset.

#### 3.2.1. Results of the Proposed End-to-End CNN Model (First Scenario)

**Figure 4:**
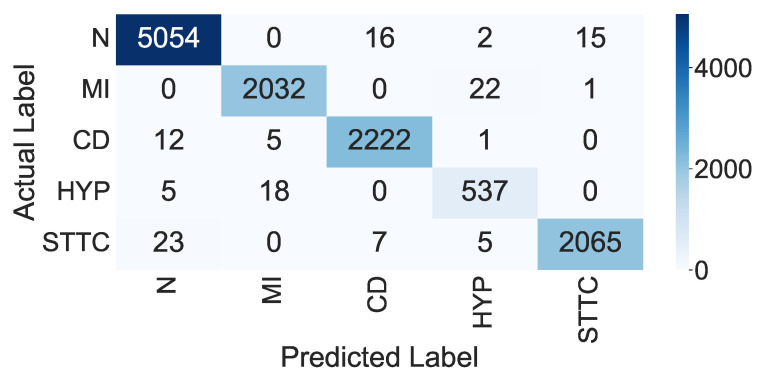
Confusion matrix of the proposed end-to-end CNN model.

**Figure 5:**
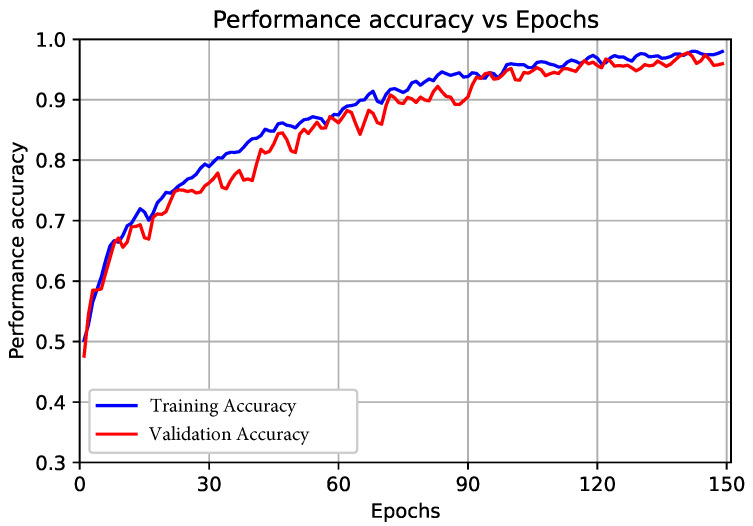
Proposed end-to-end CNN training and validation accuracy curve.

**Figure 6:**
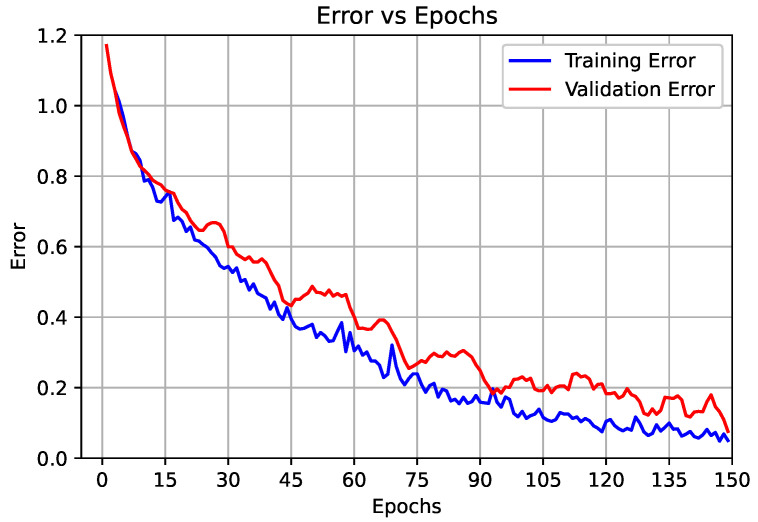
Proposed end-to-end CNN training and validation error curve.

In this scenario, we employed the deep CNN model for feature extraction and classification as an end-to-end model. The classification was performed by using the layer in the model (Soft-Max layer), where the inputs of this layer were the vector features resulting from the dense layer with a size of 1000 to classify five heart signal classes. The confusion matrix of the proposed model is shown in Figure 4. In addition, the overall performance of the proposed CNN model for each class based on the presented evaluation metrics is shown in Table 6. Moreover, the accuracy and loss curves for the training and validation are shown in Figure 5 and Figure 6, respectively.

From the confusion matrix in Figure 4, we can show that approximately 99.39% of the ECG signals were correctly classified as normal signals, 0.61% of the normal signals were wrongly classified as CD signals, 0.03% of the normal signals were wrongly classified as HPY signals, and 0.31% were wrongly classified as STTC signals. In addition, 99.62% of the MI signals were correctly classified as MI signals, 1.08% were wrongly classified as HYP signals, and 0.04% were wrongly classified as STTC signals. Moreover, 99.66% of the CD signals were correctly classified as CD signals, 0.53% were wrongly classified as normal signals, 0.22% were wrongly classified as MI signals, and 0.04% were wrongly classified as HPY signals. In addition, 99.56% of the HYP signals were correctly classified as HYP signals, 0.89% were wrongly classified as normal signals, and 3.21% were wrongly classified as MI signals. Finally, 99.57% of the STTC signals were correctly classified as STTC signals, 1.09% were wrongly classified as normal signals, 0.33% were wrongly classified as CD signals, and 0.23% were wrongly classified as HYP signals.

From the analysis of the results shown in Table 6, we can observe that the overall accuracy of the proposed end-to-end CNN model was 98.90% with a total TP equal to 11,710. The average *Pre* for all five classes was 98.20%, the average *Rec* for all five class was 98.20%, and the average *F*-score for all classes was 98.20%. From the curves in Figure 5 and Figure 6, we can observe that, after 90 epochs of training, the improvements were marginal, and after 120 epochs, the model could be considered fully trained.

#### 3.2.2. Results of the Proposed Deep CNN Model with the SVM Classifier (Second Scenario)

**Figure 7:**
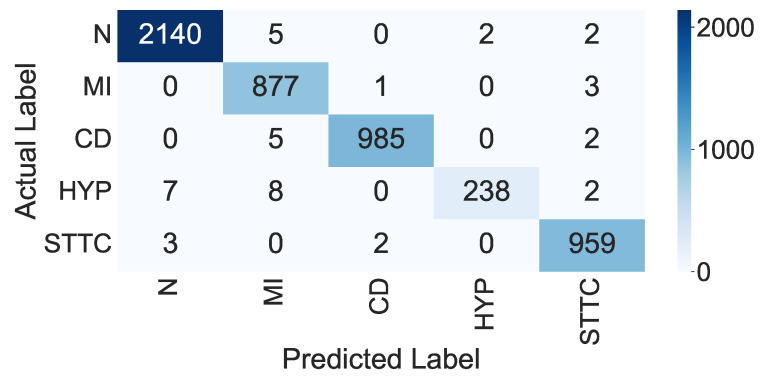
Confusion matrix of the SVM classifier.

In this scenario, we employed the proposed CNN as a feature extractor by selecting the output of the flatten layer and fed the features to an external classifier for classification (in this case, we used the SVM classifier). The flatten layer provided a 1D feature set (from Feature 1 to Feature 1000) for each ECG signal row. A multi-class SVM classifier was used in this paper to classify the used data into the five ECG classes. The confusion matrix of the proposed model when using the SVM as a classifier is shown in Figure 7. In addition, the overall performance of the CNN model with the SVM for each class based on the presented evaluation metrics is shown in Table 7.

From the confusion matrix in Figure 7, we can see that 99.64% of the normal ECG signals were correctly categorized as normal signals, 0.36% were wrongly categorized as MI signals, 0.09% were wrongly categorized as HPY signals, and 0.09% were wrongly categorized as STTC signals. In addition, 99.58% of the MI signals were correctly categorized as MI signals, 0.42% were wrongly categorized as CD signals, and 0.34% were wrongly categorized as STTC signals. Moreover, 99.81% of the CD signals were correctly categorized as CD signals, 0.50% were wrongly categorized as MI signals, and 0.20% were wrongly categorized as STTC signals. In addition, 99.64% of the HYP signals were correctly categorized as HYP signals, 2.74% were wrongly categorized as normal signals, 3.13% were wrongly categorized as MI signals, and 0.78% were wrongly categorized as STTC signals. Finally, 99.73% of the STTC signals were correctly categorized as STTC signals, 0.31% were wrongly categorized as normal signals, and 0.20% were wrongly categorized as CD signals.

From the analysis of the results shown in Table 7, we can observe that the overall accuracy of the proposed CNN model with the SVM was 99.20% with a total TP equal to 5199. The average *Pre* for all five classes was 99.09%, the average *Rec* for all five classes was 98.24%, and the average *F*-score for all classes was 98.58%.

From the previous results, we can observe that the overall performance of the proposed deep CNN model with the SVM was better than that when using the deep CNN model for classification. A comparison between the proposed deep CNN model and other recent works on the PTB-XL database is presented in Table 8. From the table, we can observe that the proposed model was more robust and efficient than other deep learning models. Therefore, in our future work, we can try new technologies for adaptive feature selection and reduction of the feature dimension, such as those in [30,31,32], to improve the performance of our method.

## 4. Conclusions

The main contribution of this study is the proposal of a new approach based on the selection of deep features for the detection of MI and CDs without using any handcrafted feature extraction methods or requiring the detection of the QRS complex. The efficiency of the proposed system was analyzed by using the PTB-XL database and was compared with existing state-of-the-art methods in this field. In addition, we proposed a new custom activation function, which was fast compared to regular activation functions, such as the sigmoid function. A ten-fold cross-validation approach was applied in order to evaluate the proposed method. Two scenarios were employed to evaluate the proposed model. The first scenario used the proposed deep CNN model for classification, and the second scenario used the CNN as a feature extractor and the SVM as a classifier to classify normal ECG signals and four other heart diseases. The results showed that the second scenario achieved better performance than those of the first scenario. The best accuracy, precision, recall, and *F*-score of the proposed model were 99.20%, 99.09%, 98.24%, and 98.58%, respectively. The proposed method was more robust and more efficient than other previous deep models for the detection of MI and CD signals. In the future, we will test the proposed deep model on several types of signals, such as electroencephalogram (EEG) signals, to see how it performs. In addition, we can employ an optimization technique, such as a genetic algorithm, to select the best features from the extracted deep features. Furthermore, adaptive selection/weighting of features is typically used for dimensionality reduction and performance improvement. The features with high discrimination, high accuracy, and low correlation should be selected and provided with high weights. Finally, we can try to employ more than one classifier (i.e., an ensemble classifier) on the same dataset and observe the results.

## Figures and Tables

**Table 1:** Comparison of related works.

Literature	Year	Database	Classifiers	Remarks (Accuracy in %)
Smigiel et al. [14]	2021	PTB-XL	CNN SincNet	72.00 73.00
Smigiel et al. [8]	2021	PTB-XL	Neural networks	76.20
Pałczynski et al. [11]	2022	PTB-XL	Neural networks	80.20
Prabhakararao et al. [12]	2022	PTB-XL CinC-training	DMSCE	84.50 88.30
Zhang et al. [13]	2021	China Physiological Signal Challenge 2018	MLBF-Net	87.70
Jahmunah et al. [15]	2021	PTB	GABORCNN	98.84
Alghamdi et al. [16]	2020	PTB	VGG-Net	99.20
Anand et al. [17]	2022	PTB-XL	CNN	95.80
He et al. [18]	2021	Combination of PTB and PTB-XL	Multi-feature-branch lead attention neural network (MFB-LANN)	94.19

**Table 2:** Original dataset without multiple annotations.

Beat Name	Number of Beats Utilized for Training and Cross-Validation	Number of Beats Utilized for Testing
Normal (Norm)	5087	2150
Myocardial infarction (MI)	2055	881
Conduction disturbance (CD)	2240	992
Hypertrophy (HYP)	560	255
ST/T change (STTC)	2100	964

**Table 3:** Detailed splitting of the data to validate the proposed method.

Total Number of Beats in the PTB-XL ECG Database	Number of Beats for Training and 10-Fold Validation (70%)	Number of Beats for Testing (30%)
17,232	12,040	5242

**Table 4:** The performance of the proposed network with various activation functions.

No	Activation Function Name	Performance in Detection of the ECG Beats (in %)
1	Sigmoid	98.46
2	tanh	96.32
3	ReLU	93.67
**4**	**Customized activation function**	**99.56**

**Table 5:** The default settings used to implement the SVM.

Hyperparameters	Values
Regularization parameter C	1.0
Kernel	`Radial Basis Function (RBF) kernel’
Degree of the polynomial kernel function	3
Gamma (kernel coefficient for `rbf’)	`scale’
Shrinking	True (if the number of iterations is large, then shrinking can shorten the training time)
Probability	False (Whether probability estimates are enabled—this must be enabled before running the fit; it will slow down that method as it utilizes 10-fold cross-validation internally, and the predict_proba may differ from predict).
Tol	0.001 (tolerance for the stopping criterion).
Cache_size	200 (specifying the size of the kernel cache).
max_iter	−1 (hard limit on iterations within the solver, or −1 for no limit).
decision_function_shape	`ovr’.
break_ties	True (if true, decision_function_shape = `ovr’ and number of classes > 2, predict will break ties based on the confidence values of decision_function; otherwise, the first tied class is returned. Please note that breaking ties incurs a somewhat large computational cost relative to a simple prediction).
random_state	None (controls the creation of pseudo-random numbers for shuffling data for probability estimates. When the probability is False, it is ignored. An integer is passed for output that is reproducible over several function calls).

**Table 6:** Overall performance of the proposed end-to-end CNN model for each class.

Class	n (Truth)	n (Classified)	Accuracy (in %)	Precision (in %)	Recall (in %)	F-Score
**N**	5054	5087	99.39	99.21	99.35	0.992
**MI**	2032	2055	99.62	98.88	98.77	0.988
**CD**	2022	2240	99.66	98.98	99.20	0.990
**HYP**	537	560	99.56	94.74	95.89	0.953
**STTC**	2065	2100	99.57	99.23	98.33	0.987
**Overall Accuracy**				98.90 (in %)		

**Table 7:** Overall performance of the proposed CNN model with the SVM for each class.

Class	n (Truth)	n (Classified)	Accuracy (in %)	Precision (in %)	Recall (in %)	F-Score
**N**	2140	2149	99.64	99.53	99.58	0.995
**MI**	877	881	99.58	97.99	99.55	0.987
**CD**	985	992	99.81	99.70	99.29	0.994
**HYP**	238	255	99.64	99.17	93.33	0.961
**STTC**	959	964	99.73	99.07	99.48	0.992
**Overall Accuracy**				99.20 (in %)		

**Table 8:** Comparison between the proposed deep CNN method and other recent methods.

Literature	Year	Database	Technique	*Acc* (in %)	*Pre* (in %)	*Rec* (in %)	F-Score
Smigiel et al. [14]	2021	PTB-XL	CNN and entropy-based features	89.14	71.40	66.20	68.00
Smigiel et al. [8]	2021	PTB-XL	Deep learning and R-peak detection	76.20	66.7	66.7	68.30
Pałczynski et al. [11]	2022	PTB-XL	Deep CNN and QRS complex detection	79.00	70.60	70.60	70.60
Prabhakararao et al. [12]	2021	PTB-XL	CNN ensemble	85.65	84.25	85.21	84.55
Zhang et al. [13]	2021	PTB-XL	Multi-lead-branch fusion network	93.10	94.30	93.10	92.80
Proposed Method	2022	PTB-XL	Deep CNN model with SVM classifier	99.20	98.20	99.20	98.60

## Data Availability

Not applicable.
